# 1-(2-Fluoro­phen­yl)-3-(3,4,5-trimethoxy­benzo­yl)thio­urea

**DOI:** 10.1107/S160053681001408X

**Published:** 2010-04-21

**Authors:** Aamer Saeed, Uzma Shaheen, Ulrich Flörke

**Affiliations:** a Department of Chemistry, Quaid-i-Azam University, Islamabad 45320, Pakistan; b Department Chemie, Fakultät für Naturwissenschaften, Universität Paderborn, Warburgerstrasse 100, D-33098 Paderborn, Germany

## Abstract

The two *m*-meth­oxy groups of the title compound, C_17_H_17_FN_2_O_4_S, are almost coplanar with the aromatic ring [CH_3_—O—C—C = 5.8 (1) and 5.9 (1)°], whereas the meth­oxy group in the *para* position is bent out of the ring plane [78.6 (1)°]. Mol­ecules are connected by inter­molecular N—H⋯S hydrogen bonds to form centrosymmetric dimers that are stacked along the *a* axis.

## Related literature

For details of the biological activity of fluorinated thio­ureas, see: Sun *et al.* (2006 ▶); Saeed *et al.* (2009 ▶); Xu *et al.* (2003 ▶). For the use of fluorinated thio­ureas in organic synthesis, see: Nosova *et al.* (2006 ▶, 2007 ▶); Berkessel *et al.* (2006 ▶). For fluorine-containing heterocycles, see: Lipunova *et al.* (2008 ▶). For intra­molecular hydrogen bonds and Fermi resonance measurements, see: Hritzová & Koščík (2008 ▶).
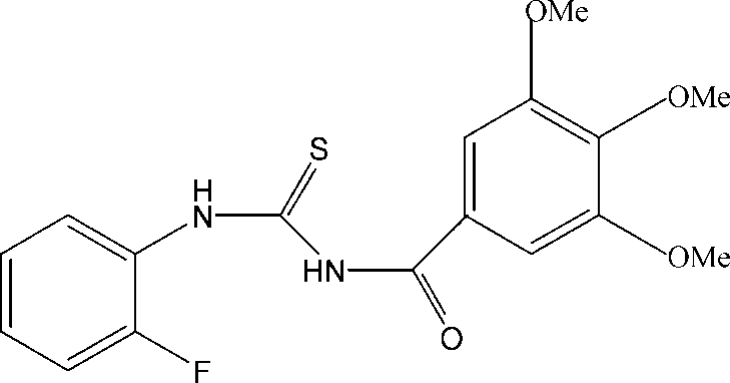


## Experimental

### 

#### Crystal data

C_17_H_17_FN_2_O_4_S
*M* *_r_* = 364.39
Triclinic, 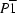

*a* = 4.0828 (5) Å
*b* = 14.0420 (16) Å
*c* = 14.2295 (16) Å
α = 91.092 (2)°
β = 90.694 (2)°
γ = 91.712 (2)°
*V* = 815.21 (16) Å^3^
*Z* = 2
Mo *K*α radiation
μ = 0.24 mm^−1^
*T* = 120 K
0.48 × 0.20 × 0.19 mm

#### Data collection

Bruker SMART APEX diffractometer
Absorption correction: multi-scan (*SADABS*; Bruker, 2002 ▶) *T*_min_ = 0.896, *T*_max_ = 0.957
7686 measured reflections
3868 independent reflections
3128 reflections with *I* > 2σ(*I*)
*R*_int_ = 0.027

#### Refinement

*R*[*F* ^2^ > 2σ(*F* ^2^)] = 0.042
*wR*(*F* ^2^) = 0.109
*S* = 1.03
3868 reflections
226 parameters
H-atom parameters constrained
Δρ_max_ = 0.36 e Å^−3^
Δρ_min_ = −0.27 e Å^−3^

### 

Data collection: *SMART* (Bruker, 2002 ▶); cell refinement: *SAINT* (Bruker, 2002 ▶); data reduction: *SAINT*; program(s) used to solve structure: *SHELXS97* (Sheldrick, 2008 ▶); program(s) used to refine structure: *SHELXL97* (Sheldrick, 2008 ▶); molecular graphics: *SHELXTL* (Sheldrick, 2008 ▶); software used to prepare material for publication: *SHELXL97*.

## Supplementary Material

Crystal structure: contains datablocks global, I. DOI: 10.1107/S160053681001408X/si2257sup1.cif

Structure factors: contains datablocks I. DOI: 10.1107/S160053681001408X/si2257Isup2.hkl

Additional supplementary materials:  crystallographic information; 3D view; checkCIF report

## Figures and Tables

**Table 1:** Hydrogen-bond geometry (Å, °)

*D*—H⋯*A*	*D*—H	H⋯*A*	*D*⋯*A*	*D*—H⋯*A*
N2—H2*A*⋯S1^i^	0.88	2.70	3.5219 (15)	157

Symmetry code: (i) 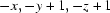.

**Fig. 1.**
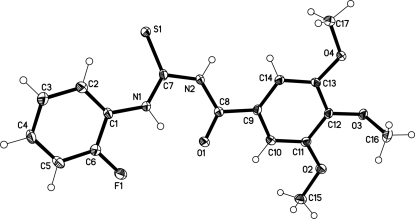
Molecular structure of the title compound. Displacement ellipsoids are drawn at the 50% probability level.

**Fig. 2.**
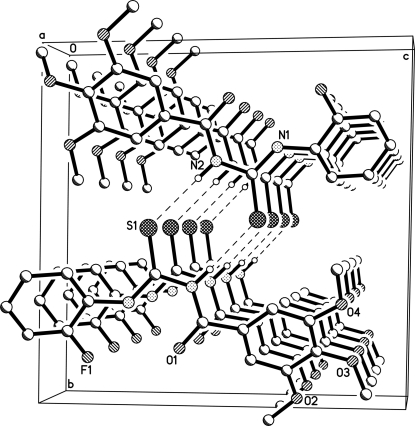
Crystal packing viewed along [100] with intermolecular hydrogen bonds indicated as dashed lines. H-atoms not involved in hydrogen bonding are omitted.

## supplementary crystallographic information

### Comment

Fluorinated thioureas are convenient synthons for preparation of versatile fluorine-containing heterocycles: [1,3]-benzothiazin-4-ones (Nosova *et al.*,2006, 2007). 1-aryl-2-ethylthio-quinazolin-4-one, thiazolidine and 1*H*-1,2,4-triazoles (Lipunova *et al.*, 2008). These constitute a novel class of potent influenza virus neuraminidase inhibitors (Sun *et al.*, 2006). Fluorinated bis-thiourea derivatives are used as organocatalyst in Morita-Baylis-Hillman reaction (Berkessel *et al.*, 2006). N-Substituted *N*'-(2-fluorobenzoyl)thiourea derivatives are suitable substrates for studying Intramolecular Hydrogen Bonds and Fermi Resonance (Hritzová & Koščík 2008). Fluorinated thioureas have shown potent microbial (Saeed *et al.*, 2009) and insecticidal activities (Xu *et al.*, 2003). The two *m*-methoxy groups of the title compound, (Fig. 1), C_17_H_17_FN_2_O_4_S, are almost coplanar with the aromatic ring [CH_3_—O—C—C 5.8 (1)° and 5.9 (1)°] whereas the methoxygroup in *para* position is bent out of the ring plane [78.6 (1)°]. The molecules are connected by intermolecular N—H···S hydrogen bonds (Table 1) to centrosymmetric dimers that are stacked along the a axis (Fig. 2).

### Experimental

3,4,5-Trimethoxybenzoylisothiocyante (1 mmol) in acetone w as treated with 2-fluoroaniline (1 mmol) under a nitrogen atmosphere at reflux for 2.5 h. Upon cooling, the reaction mixture was poured into aq HCl and the precipitated product was rerystallized from in methanol to afforded the title compound (86 %) as colourless crystals: Anal. calcd. for C_17_H_17_N_2_O_4_F_2_S: C, 56.03; H, 4.70; N, 7.69; S, 8.80%; found: C, 56.12; H, 4.76; N, 7.71; S, 8.76%.

### Refinement

Hydrogen atoms were clearly identified in difference Fourier syntheses, idealized and refined at calculated positions riding on the carbon atoms with isotropic displacement parameters *U*_iso_(H) = 1.2*U*_eq_(C/N) or 1.5*U*_eq_(-CH_3_). All methyl H atoms were allowed to rotate but not to tip.

### Crystal data

**Table:**

C_17_H_17_FN_2_O_4_S	*Z* = 2
*M**_r_* = 364.39	*F*(000) = 380
Triclinic, *P*1	*D*_x_ = 1.484 Mg m^−^^3^
Hall symbol: -P 1	Mo *K*α radiation, λ = 0.71073 Å
*a* = 4.0828 (5) Å	Cell parameters from 2067 reflections
*b* = 14.0420 (16) Å	θ = 2.9–28.2°
*c* = 14.2295 (16) Å	µ = 0.24 mm^−^^1^
α = 91.092 (2)°	*T* = 120 K
β = 90.694 (2)°	Prism, colourless
γ = 91.712 (2)°	0.48 × 0.20 × 0.19 mm
*V* = 815.21 (16) Å^3^	

### Data collection

**Table:**

Bruker SMART APEX diffractometer	3868 independent reflections
Radiation source: sealed tube	3128 reflections with *I* > 2σ(*I*)
graphite	*R*_int_ = 0.027
φ and ω scans	θ_max_ = 27.9°, θ_min_ = 1.4°
Absorption correction: multi-scan (*SADABS*; Bruker, 2002)	*h* = −5→5
*T*_min_ = 0.896, *T*_max_ = 0.957	*k* = −18→18
7686 measured reflections	*l* = −17→18

### Refinement

**Table:**

Refinement on *F*^2^	Primary atom site location: structure-invariant direct methods
Least-squares matrix: full	Secondary atom site location: difference Fourier map
*R*[*F*^2^ > 2σ(*F*^2^)] = 0.042	Hydrogen site location: difference Fourier map
*wR*(*F*^2^) = 0.109	H-atom parameters constrained
*S* = 1.03	*w* = 1/[σ^2^(*F*_o_^2^) + (0.0485*P*)^2^ + 0.2731*P*] where *P* = (*F*_o_^2^ + 2*F*_c_^2^)/3
3868 reflections	(Δ/σ)_max_ < 0.001
226 parameters	Δρ_max_ = 0.36 e Å^−^^3^
0 restraints	Δρ_min_ = −0.27 e Å^−^^3^

### Special details

**Table:**

Geometry. All esds (except the esd in the dihedral angle between two l.s. planes) are estimated using the full covariance matrix. The cell esds are taken into account individually in the estimation of esds in distances, angles and torsion angles; correlations between esds in cell parameters are only used when they are defined by crystal symmetry. An approximate (isotropic) treatment of cell esds is used for estimating esds involving l.s. planes.
Refinement. Refinement of *F*^2^ against ALL reflections. The weighted *R*-factor wR and goodness of fit *S* are based on *F*^2^, conventional *R*-factors *R* are based on *F*, with *F* set to zero for negative *F*^2^. The threshold expression of *F*^2^ > σ(*F*^2^) is used only for calculating *R*-factors(gt) etc. and is not relevant to the choice of reflections for refinement. *R*-factors based on *F*^2^ are statistically about twice as large as those based on *F*, and *R*- factors based on ALL data will be even larger.

### Fractional atomic coordinates and isotropic or equivalent isotropic displacement parameters (Å^2^)

**Table:**

	*x*	*y*	*z*	*U*_iso_*/*U*_eq_	
S1	0.28840 (12)	0.51052 (3)	0.37700 (3)	0.02191 (13)	
F1	0.1367 (3)	0.83707 (8)	0.21822 (8)	0.0346 (3)	
O1	0.0156 (4)	0.81511 (9)	0.44315 (9)	0.0287 (3)	
O2	−0.5433 (3)	0.95757 (8)	0.72291 (8)	0.0239 (3)	
O3	−0.3506 (3)	0.85284 (8)	0.86784 (8)	0.0220 (3)	
O4	0.0054 (3)	0.70067 (8)	0.84415 (8)	0.0228 (3)	
N1	0.2791 (4)	0.69556 (10)	0.32920 (10)	0.0220 (3)	
H1A	0.2360	0.7532	0.3500	0.026*	
N2	0.0880 (4)	0.65816 (10)	0.47587 (10)	0.0199 (3)	
H2A	0.0559	0.6148	0.5189	0.024*	
C1	0.3979 (4)	0.69150 (12)	0.23660 (12)	0.0199 (4)	
C2	0.5894 (5)	0.62118 (13)	0.19798 (13)	0.0264 (4)	
H2B	0.6512	0.5687	0.2347	0.032*	
C3	0.6906 (5)	0.62772 (13)	0.10527 (14)	0.0309 (5)	
H3A	0.8210	0.5792	0.0791	0.037*	
C4	0.6043 (5)	0.70389 (13)	0.05025 (13)	0.0279 (4)	
H4A	0.6729	0.7071	−0.0132	0.033*	
C5	0.4183 (5)	0.77486 (13)	0.08855 (13)	0.0271 (4)	
H5A	0.3590	0.8279	0.0522	0.032*	
C6	0.3206 (5)	0.76752 (12)	0.17987 (13)	0.0232 (4)	
C7	0.2210 (4)	0.62634 (12)	0.39079 (11)	0.0176 (3)	
C8	0.0018 (4)	0.74974 (11)	0.49981 (12)	0.0190 (3)	
C9	−0.1053 (4)	0.76904 (11)	0.59750 (11)	0.0174 (3)	
C10	−0.2858 (4)	0.85088 (11)	0.61089 (12)	0.0189 (3)	
H10A	−0.3518	0.8868	0.5584	0.023*	
C11	−0.3685 (4)	0.87953 (11)	0.70090 (12)	0.0187 (3)	
C12	−0.2668 (4)	0.82639 (11)	0.77787 (11)	0.0182 (3)	
C13	−0.0860 (4)	0.74466 (11)	0.76356 (11)	0.0175 (3)	
C14	−0.0055 (4)	0.71485 (11)	0.67339 (12)	0.0181 (3)	
H14A	0.1150	0.6588	0.6635	0.022*	
C15	−0.6232 (5)	1.01977 (12)	0.64767 (13)	0.0262 (4)	
H15A	−0.7484	1.0730	0.6723	0.039*	
H15B	−0.7549	0.9843	0.5999	0.039*	
H15C	−0.4210	1.0444	0.6194	0.039*	
C16	−0.1525 (5)	0.93050 (14)	0.90583 (13)	0.0296 (4)	
H16A	−0.2241	0.9460	0.9697	0.044*	
H16B	−0.1753	0.9863	0.8662	0.044*	
H16C	0.0774	0.9124	0.9077	0.044*	
C17	0.1706 (5)	0.61312 (12)	0.83456 (13)	0.0247 (4)	
H17B	0.2231	0.5891	0.8970	0.037*	
H17C	0.3735	0.6237	0.7996	0.037*	
H17D	0.0289	0.5663	0.8004	0.037*	

### Atomic displacement parameters (Å^2^)

**Table:**

	*U*^11^	*U*^22^	*U*^33^	*U*^12^	*U*^13^	*U*^23^
S1	0.0321 (3)	0.0160 (2)	0.0179 (2)	0.00099 (17)	0.00664 (18)	0.00108 (16)
F1	0.0514 (8)	0.0262 (6)	0.0278 (6)	0.0146 (5)	0.0146 (5)	0.0083 (5)
O1	0.0488 (9)	0.0200 (6)	0.0181 (6)	0.0067 (6)	0.0106 (6)	0.0048 (5)
O2	0.0307 (7)	0.0210 (6)	0.0207 (6)	0.0083 (5)	0.0058 (5)	0.0027 (5)
O3	0.0309 (7)	0.0197 (6)	0.0152 (6)	−0.0016 (5)	0.0066 (5)	−0.0027 (5)
O4	0.0358 (8)	0.0189 (6)	0.0139 (6)	0.0060 (5)	0.0014 (5)	0.0024 (5)
N1	0.0360 (9)	0.0155 (7)	0.0147 (7)	0.0027 (6)	0.0068 (6)	0.0006 (5)
N2	0.0310 (9)	0.0165 (7)	0.0123 (7)	0.0005 (6)	0.0037 (6)	0.0016 (5)
C1	0.0266 (10)	0.0195 (8)	0.0135 (8)	−0.0010 (7)	0.0035 (7)	0.0018 (6)
C2	0.0348 (11)	0.0218 (9)	0.0233 (9)	0.0045 (8)	0.0084 (8)	0.0050 (7)
C3	0.0431 (12)	0.0252 (9)	0.0250 (10)	0.0035 (8)	0.0141 (9)	−0.0003 (8)
C4	0.0388 (12)	0.0298 (10)	0.0150 (8)	−0.0035 (8)	0.0076 (8)	0.0017 (7)
C5	0.0339 (11)	0.0277 (9)	0.0199 (9)	−0.0004 (8)	0.0022 (8)	0.0091 (7)
C6	0.0281 (10)	0.0208 (8)	0.0209 (9)	0.0028 (7)	0.0047 (7)	0.0004 (7)
C7	0.0211 (9)	0.0196 (8)	0.0122 (8)	−0.0004 (6)	0.0017 (6)	−0.0002 (6)
C8	0.0241 (9)	0.0174 (8)	0.0156 (8)	0.0001 (6)	0.0023 (7)	0.0007 (6)
C9	0.0215 (9)	0.0160 (7)	0.0146 (8)	−0.0031 (6)	0.0038 (7)	−0.0019 (6)
C10	0.0228 (9)	0.0175 (8)	0.0167 (8)	−0.0003 (6)	0.0027 (7)	0.0036 (6)
C11	0.0201 (9)	0.0159 (8)	0.0202 (8)	−0.0004 (6)	0.0033 (7)	0.0000 (6)
C12	0.0233 (9)	0.0167 (8)	0.0146 (8)	−0.0024 (6)	0.0046 (7)	−0.0016 (6)
C13	0.0221 (9)	0.0150 (7)	0.0153 (8)	−0.0024 (6)	0.0004 (7)	0.0032 (6)
C14	0.0224 (9)	0.0141 (7)	0.0176 (8)	−0.0008 (6)	0.0029 (7)	−0.0002 (6)
C15	0.0312 (11)	0.0219 (9)	0.0260 (10)	0.0063 (7)	0.0017 (8)	0.0053 (7)
C16	0.0355 (11)	0.0311 (10)	0.0216 (9)	−0.0034 (8)	0.0032 (8)	−0.0087 (8)
C17	0.0320 (10)	0.0207 (8)	0.0217 (9)	0.0059 (7)	−0.0006 (8)	0.0031 (7)

### Geometric parameters (Å, °)

**Table:**

S1—C7	1.6659 (17)	C4—H4A	0.9500
F1—C6	1.360 (2)	C5—C6	1.369 (2)
O1—C8	1.2337 (19)	C5—H5A	0.9500
O2—C11	1.359 (2)	C8—C9	1.485 (2)
O2—C15	1.435 (2)	C9—C10	1.395 (2)
O3—C12	1.3763 (19)	C9—C14	1.397 (2)
O3—C16	1.432 (2)	C10—C11	1.384 (2)
O4—C13	1.3660 (19)	C10—H10A	0.9500
O4—C17	1.425 (2)	C11—C12	1.403 (2)
N1—C7	1.339 (2)	C12—C13	1.396 (2)
N1—C1	1.410 (2)	C13—C14	1.388 (2)
N1—H1A	0.8800	C14—H14A	0.9500
N2—C8	1.381 (2)	C15—H15A	0.9800
N2—C7	1.404 (2)	C15—H15B	0.9800
N2—H2A	0.8800	C15—H15C	0.9800
C1—C2	1.387 (2)	C16—H16A	0.9800
C1—C6	1.392 (2)	C16—H16B	0.9800
C2—C3	1.391 (3)	C16—H16C	0.9800
C2—H2B	0.9500	C17—H17B	0.9800
C3—C4	1.389 (3)	C17—H17C	0.9800
C3—H3A	0.9500	C17—H17D	0.9800
C4—C5	1.379 (3)		
			
C11—O2—C15	117.24 (13)	C14—C9—C8	122.59 (15)
C12—O3—C16	113.37 (13)	C11—C10—C9	119.76 (15)
C13—O4—C17	117.45 (13)	C11—C10—H10A	120.1
C7—N1—C1	130.78 (14)	C9—C10—H10A	120.1
C7—N1—H1A	114.6	O2—C11—C10	125.26 (15)
C1—N1—H1A	114.6	O2—C11—C12	115.17 (14)
C8—N2—C7	127.44 (14)	C10—C11—C12	119.56 (15)
C8—N2—H2A	116.3	O3—C12—C13	119.38 (14)
C7—N2—H2A	116.3	O3—C12—C11	120.47 (15)
C2—C1—C6	117.45 (16)	C13—C12—C11	120.13 (15)
C2—C1—N1	126.57 (15)	O4—C13—C14	124.86 (15)
C6—C1—N1	115.96 (15)	O4—C13—C12	114.50 (14)
C1—C2—C3	119.80 (17)	C14—C13—C12	120.63 (15)
C1—C2—H2B	120.1	C13—C14—C9	118.61 (15)
C3—C2—H2B	120.1	C13—C14—H14A	120.7
C4—C3—C2	121.17 (18)	C9—C14—H14A	120.7
C4—C3—H3A	119.4	O2—C15—H15A	109.5
C2—C3—H3A	119.4	O2—C15—H15B	109.5
C5—C4—C3	119.40 (17)	H15A—C15—H15B	109.5
C5—C4—H4A	120.3	O2—C15—H15C	109.5
C3—C4—H4A	120.3	H15A—C15—H15C	109.5
C6—C5—C4	118.79 (16)	H15B—C15—H15C	109.5
C6—C5—H5A	120.6	O3—C16—H16A	109.5
C4—C5—H5A	120.6	O3—C16—H16B	109.5
F1—C6—C5	119.23 (15)	H16A—C16—H16B	109.5
F1—C6—C1	117.40 (15)	O3—C16—H16C	109.5
C5—C6—C1	123.38 (17)	H16A—C16—H16C	109.5
N1—C7—N2	114.10 (14)	H16B—C16—H16C	109.5
N1—C7—S1	127.56 (13)	O4—C17—H17B	109.5
N2—C7—S1	118.34 (12)	O4—C17—H17C	109.5
O1—C8—N2	122.03 (15)	H17B—C17—H17C	109.5
O1—C8—C9	119.80 (15)	O4—C17—H17D	109.5
N2—C8—C9	118.16 (14)	H17B—C17—H17D	109.5
C10—C9—C14	121.30 (15)	H17C—C17—H17D	109.5
C10—C9—C8	115.80 (14)		
			
C7—N1—C1—C2	−24.6 (3)	C14—C9—C10—C11	0.0 (3)
C7—N1—C1—C6	157.28 (18)	C8—C9—C10—C11	−173.74 (15)
C6—C1—C2—C3	−1.2 (3)	C15—O2—C11—C10	−5.8 (3)
N1—C1—C2—C3	−179.34 (19)	C15—O2—C11—C12	173.60 (15)
C1—C2—C3—C4	0.2 (3)	C9—C10—C11—O2	−179.91 (16)
C2—C3—C4—C5	0.8 (3)	C9—C10—C11—C12	0.7 (3)
C3—C4—C5—C6	−0.7 (3)	C16—O3—C12—C13	102.98 (18)
C4—C5—C6—F1	−179.93 (17)	C16—O3—C12—C11	−78.6 (2)
C4—C5—C6—C1	−0.3 (3)	O2—C11—C12—O3	1.5 (2)
C2—C1—C6—F1	−179.10 (16)	C10—C11—C12—O3	−179.01 (15)
N1—C1—C6—F1	−0.8 (3)	O2—C11—C12—C13	179.89 (15)
C2—C1—C6—C5	1.3 (3)	C10—C11—C12—C13	−0.6 (3)
N1—C1—C6—C5	179.62 (18)	C17—O4—C13—C14	−5.9 (2)
C1—N1—C7—N2	−177.33 (17)	C17—O4—C13—C12	175.70 (15)
C1—N1—C7—S1	2.7 (3)	O3—C12—C13—O4	−3.2 (2)
C8—N2—C7—N1	2.9 (3)	C11—C12—C13—O4	178.39 (15)
C8—N2—C7—S1	−177.17 (14)	O3—C12—C13—C14	178.27 (15)
C7—N2—C8—O1	4.8 (3)	C11—C12—C13—C14	−0.1 (3)
C7—N2—C8—C9	−174.22 (16)	O4—C13—C14—C9	−177.53 (16)
O1—C8—C9—C10	20.7 (3)	C12—C13—C14—C9	0.8 (3)
N2—C8—C9—C10	−160.21 (16)	C10—C9—C14—C13	−0.8 (3)
O1—C8—C9—C14	−152.96 (18)	C8—C9—C14—C13	172.57 (16)
N2—C8—C9—C14	26.1 (3)		

### Hydrogen-bond geometry (Å, °)

**Table:**

*D*—H···*A*	*D*—H	H···*A*	*D*···*A*	*D*—H···*A*
N2—H2A···S1^i^	0.88	2.70	3.5219 (15)	157

Symmetry codes: (i) −*x*, −*y*+1, −*z*+1.
